# Performance of amide proton transfer imaging to differentiate true progression from therapy-related changes in gliomas and metastases

**DOI:** 10.1007/s00330-024-11004-y

**Published:** 2024-08-12

**Authors:** Rajeev A. Essed, Yeva Prysiazhniuk, Ivar J. Wamelink, Aynur Azizova, Vera C. Keil

**Affiliations:** 1 Department of Radiology and Nuclear Medicine, Amsterdam University Medical Center, De Boelelaan 1117, 1081HV Amsterdam, Netherlands; 2 Charles University, The Second Faculty of Medicine, Department of Pathophysiology, Prague, Czech Republic; 3 Cancer Center Amsterdam, Imaging and Biomarkers, De Boelelaan 1117, 1081HV Amsterdam, Netherlands; 4 Amsterdam Neuroscience, Brain Imaging, De Boelelaan 1117, 1081HV Amsterdam, Netherlands

**Keywords:** Molecular imaging, Magnetic resonance imaging, Therapy response, Glioma, Brain metastasis

## Abstract

**Objectives:**

Differentiating true progression or recurrence (TP/TR) from therapy-related changes (TRC) is complex in brain tumours. Amide proton transfer-weighted (APT) imaging is a chemical exchange saturation transfer (CEST) MRI technique that may improve diagnostic accuracy during radiological follow-up. This systematic review and meta-analysis elucidated the level of evidence and details of state-of-the-art imaging for APT-CEST in glioma and brain metastasis surveillance.

**Methods:**

PubMed, EMBASE, Web of Science, and Cochrane Library were systematically searched for original articles about glioma and metastasis patients who received APT-CEST imaging for suspected TP/TR within 2 years after (chemo)radiotherapy completion. Modified Quality Assessment of Diagnostic Accuracy Studies-2 criteria were applied. A meta-analysis was performed to pool results and to compare subgroups.

**Results:**

Fifteen studies were included for a narrative synthesis, twelve of which (500 patients) were deemed sufficiently homogeneous for a meta-analysis. Magnetisation transfer ratio asymmetry performed well in gliomas (sensitivity 0.88 [0.82–0.92], specificity 0.84 [0.72–0.91]) but not in metastases (sensitivity 0.64 [0.38–0.84], specificity 0.56 [0.33–0.77]). APT-CEST combined with conventional/advanced MRI rendered 0.92 [0.86–0.96] and 0.88 [0.72–0.95] in gliomas. Tumour type, TR prevalence, sex, and acquisition protocol were sources of significant inter-study heterogeneity in sensitivity (*I*^2^ = 62.25%; *p* < 0.01) and specificity (*I*^2^ = 66.31%; *p* < 0.001).

**Conclusion:**

A growing body of literature suggests that APT-CEST is a promising technique for improving the discrimination of TP/TR from TRC in gliomas, with limited data on metastases.

**Clinical relevance statement:**

This meta-analysis identified a utility for APT-CEST imaging regarding the non-invasive discrimination of brain tumour progression from therapy-related changes, providing a critical evaluation of sequence parameters and cut-off values, which can be used to improve response assessment and patient outcome.

**Key Points:**

- *Therapy-related changes mimicking progression complicate brain tumour treatment*.
- *Amide proton imaging improves the non-invasive discrimination of glioma progression from therapy-related changes*.
- *Magnetisation transfer ratio asymmetry measurement seems not to have added value in brain metastases*.

## Introduction

Brain tumour surveillance combines follow-up magnetic resonance imaging (MRI) and clinical evaluation. The treatment of glial tumours and brain metastases (BM) involving chemoradiotherapy (CRT) or radiotherapy (RT) alone can result in radiological phenomena mimicking true tumour progression (TP) or recurrence (TR) [1]. These phenomena, sometimes coined ‘therapy-related changes’ (TRC), mainly comprise pseudoprogression (PsP) and radiation necrosis (RN) [2]. TRC misidentified as TP/TR can unnecessarily prompt treatment alterations and reduce the quality of life and overall survival [1].

The gold standard for TRC identification is histopathological assessment [3]. Pathological verification, however, conveys the invasiveness physicians seek to avoid. Neuro-oncological standard MRI protocols are frequently extended by dynamic susceptibility contrast (DSC) or arterial spin labelling (ASL) sequences, types of perfusion-weighted imaging (PWI), to discriminate TP/TR from TRC by variable cut-off values [4–6]. DSC requires gadolinium-based contrast injection and is prone to artefacts in post-therapeutic tissue due to the magnetic susceptibility of necrotic products [7]. ASL suffers from lower signal-to-noise ratios and has a lower degree of clinical validation. These circumstances highlight the need for a reliable, non-invasive, method for TRC identification.

Amide proton transfer (APT) is a type of chemical exchange saturation transfer (CEST) imaging and a relatively new technique in the MRI armamentarium to identify protein content [8, 9]. APT-CEST generates a contrast through an off-resonance saturation pulse, inducing transfer from saturated amide protons of mobile endogenous proteins to surrounding water. Most prominent is the magnetisation transfer ratio asymmetry analysis at 3.5 parts per million (MTR_asym_(3.5 ppm)), having become the first commercial APT-weighted sequence on clinical MRI scanners [10]. The non-invasive nature of APT-CEST makes it a synergistic addition to PWI that can help reduce unnecessary operations in suspected TP/TR cases. It is also less costly and invasive than another technique applied for this purpose: positron emission tomography (PET). APT-related publications for therapy response assessment in brain tumours were scarce until recently and limited to gliomas [3, 11–14]. The topic gained scientific momentum in the past 2 years, necessitating a to-date summary evaluation including both glioma and brain metastasis (BM) patients. Such an analysis is relevant for deciding if APT-CEST is a worthwhile technique to investigate in more extensive clinical studies.

This systematic review and meta-analysis aims to provide a comprehensive evaluation of the diagnostic performance of APT imaging in differentiating TP/TR from TRC in glioma and BM patients. Additionally, it presents an overview of APT imaging parameters to facilitate the harmonisation of future research efforts and lay some groundwork for clinical implementation.

## Methods

### Protocol and registration

This systematic review was registered on PROSPERO (CRD42024501112) and conducted according to the preferred reporting items for systematic review and meta-analysis of diagnostic test accuracy studies (PRISMA-DTA) [15]. One reviewer (R.E.) performed search, screening and selection, data extraction, quality/risk of bias screening, and analysis under supervision by an experienced neuroradiologist (V.K.). Another reviewer (A.A.), blinded to the quality screening of the first reviewer, performed a second screening. Disagreements were resolved through consensus discussions.

### Search strategy

PubMed, EMBASE, Web of Science, and Cochrane Library were systematically searched using a block search strategy which included three aspects: (1) gliomas and BM, (2) APT-CEST and (3) therapy response assessment (TP/TR versus TRC). The search was performed on January 12, 2024, and the search strings are provided in the supplementary material.

### Eligibility and selection

Eligibility criteria were as follows: (1) patients with pathologically proven glioma or BM, (2) who underwent CRT or RT with or without prior surgical resection, and (3) received, among other imaging, APT-CEST for (4) suspected TP/TR on follow-up.

Inclusion criteria were as follows: (1) patients with adult-type gliomas or BM, (2) assessment on APT-CEST within 2 years after (chemo)radiotherapy completion, (3) APT or multi-parametric MRI including APT-CEST, (4) histopathological or clinical-radiological assessment of therapy response as reference standard.

Exclusion criteria were as follows: (1) age < 18 years; (2) APT-CEST not defined as a means of differentiating TP or TR from TRC; (3) imaging on < 3 Tesla (T) hardware; (4) animal and ex vivo studies; (5) treatment does not include RT/CRT; (6) case series, conference abstracts or book sections; (7) non-English-language articles.

The resulting records were screened by title and abstract and underwent full-text screening based on eligibility, inclusion, and exclusion criteria for inclusion in the systematic review by one reviewer (R.E). Studies assessing MTR_asym_(3.5 ppm) performance that had data presentations allowing the determination of confusion matrices or diagnostic performance estimates were included for meta-analysis.

### Data extraction

Data extraction included study design, patient and tumour characteristics (number of patients, tumour type, tumour grade, sex, age, and cohort source to identify potential cohort overlap), intervention details (i.e., therapy, time between therapy completion and imaging), MR sequence characteristics and APT-CEST parameters (hardware, saturation pulse parameters, readout, number of offset resonance frequencies, post-processing method), reference standard details (method and follow-up period), region of interest selection, outcome details (histogram parameters and signal intensity in TP/TR versus TRC, TP/TR prevalence), and diagnostic performance metrics (area under the curve (AUC), sensitivity and specificity).

Absent parameters were inferred from the provided data if feasible. Articles were excluded from the meta-analysis if the necessary data to construct diagnostic performance contingency tables could not be extracted.

### Risk of bias and applicability

Risk of bias and applicability were assessed using a modified version of the ‘Quality Assessment of Diagnostic Accuracy Studies-2’ (QUADAS-2) tool [16]. This included the following domains: (1) patient and selection, (2) index test(s), (3) reference standard(s) and (4) flow, timing, and analysis. The modified QUADAS-2 tool is documented in the supplementary material.

### Synthesis and analysis

Textual narrative synthesis was used for qualitative data assessment. If studies had overlapping cohorts, the most methodologically similar studies with regard to a clinical assessor (e.g., no radiomics or learning-based approaches) were included for meta-analysis. If studies reported multiple histogram parameters, the best-performing one was selected for meta-analysis. Forest plot and summary receiver operating characteristics (SROC) analyses were performed to investigate the diagnostic performance of APT-CEST and multi-parametric MRI, including APT-CEST using *midas* and *metandi* modules in Stata 17 (Stata Corp.). These can help to define a benchmark for diagnostic accuracy for future studies as well as provide an indicator of added value in clinical use in comparison with other techniques. ROC analysis of signal distributions was performed using SPSS 28 if the required diagnostic performance metrics or confusion matrices were not reported. Heterogeneity was assessed using Cochrane Q and Higgins *I*^2^ tests, where *p* < 0.05 and *I*^2^ > 50% were considered significant. Univariate meta-regression and subgroup analyses were performed using the *midas* module in Stata. Subgroups were compared through likelihood ratio testing, and bivariate model parameters were extracted using the *meqrlogit* command in Stata. Finally, individual AUCs were pooled using the *psfmi* package in R.

## Results

### Study selection

**Fig. 1:**
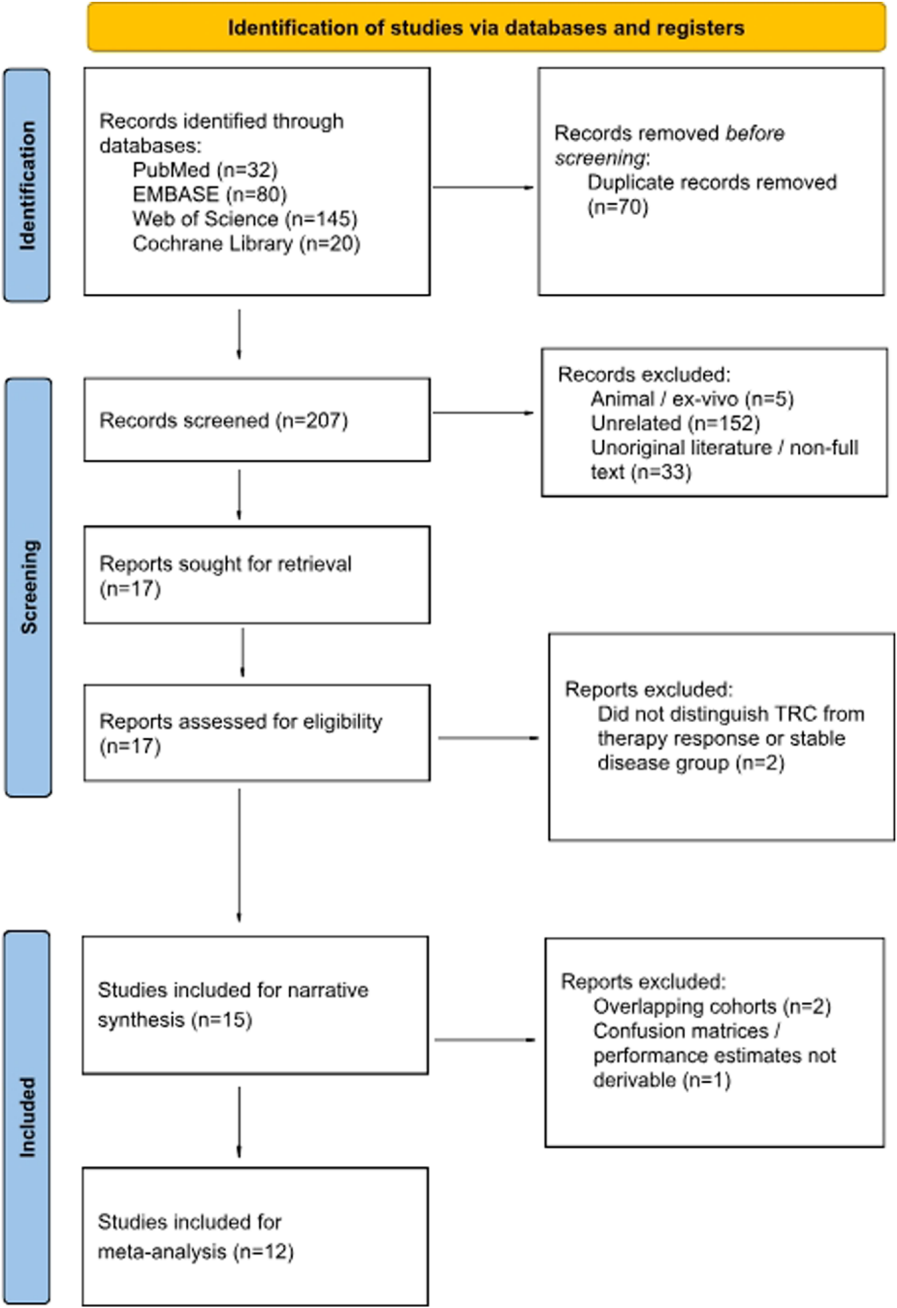
Flow diagram describing the study screening and selection

The database search yielded 277 records, of which 70 duplicates were removed (Fig. 1); 190 articles were excluded due to a misfit in the inclusion/exclusion criteria. The full texts of the remaining 17 articles were screened, excluding another two for not distinguishing TRC from stable patients or therapy responders. Finally, 15 articles were included for a narrative synthesis, three of which were excluded from the meta-analysis because cohorts potentially overlapped or data presentations did not enable confusion matrix construction, leaving twelve articles for a meta-analysis.

### Overview

Of the 15 included studies, 2 [17, 18] exclusively used APT-CEST in combination with other imaging, 6 [19–24] applied APT-CEST-only as well as combined models, while 7 [25–31] used APT-CEST-only models. Thirteen studies [17–27, 30, 31] included glioma patients, while only two [28, 29] included BM patients. One glioma study [18] used a learning-based analysis model to identify TR, and another [21] applied a radiomics analysis, both of which included cohorts from two previous studies [25, 27]. All patients, except for four in one study [17], received either CRT or RT with or without prior surgery before assessment on APT-CEST. The female patient percentage ranged from 23.8 to 68%, and patients with TP/TR ranged from 30 to 88%. The two BM studies had the highest TRC percentage and the highest female percentage among all studies. The supplementary material provides an overview of the study characteristics, including how the diagnosis was established.

### Imaging parameters

All 15 studies used a 3-T MRI scanner with a duty cycle saturation of at least 50%. The APT-weighted signal across all studies was calculated as _an MTRasym_(3.5 ppm) signal, and three studies further separated signal components, utilising Z-spectral fitting or relaxation-compensated approaches [26, 28, 29]. 3D gradient- and spin-echo was the most used imaging readout, and a water saturation shift referencing map was most used for magnetic field (B_0_) inhomogeneity correction. The supplementary material provides an overview of the sequence parameters for APT-CEST acquisition.

### APT signal and diagnostic performance

Twelve studies [17–25, 27, 30, 31] reported significantly higher APT signals in TP/TR compared to the TRC. A ROC analysis in BM [28, 29] found that the magnetisation transfer ratio of amide (MTR_Amide_), the nuclear Overhauser effect (MTR_NOE_), and relaxation-compensated APT were significantly able to distinguish TP/TR from TRC. Various histogram parameters were used to assess APT signal intensity, including APTmax [19, 20, 27, 31], APTmean [20, 22, 24–29], and APT90 [20, 23, 30, 31] (see Table 1).

**Table 1:** Overview of the histogram parameter thresholds used for magnetisation transfer ratio asymmetry (amide proton transfer-weighted) imaging

Study	Tumour	Histogram parameter	Threshold
Guo et al [18]	Glioma WHO III-IV	-	-
Hou et al [19]	Glioma WHO III-IV	APTmax*	2.25%
Huang et al [20]	Glioma WHO III-IV	APTmean*	-
		APTmax*	-
		APT90*	-
Jiang et al [25]	Glioma WHO III-IV	APTmean*	1.79%
Jiang et al [21]	Glioma WHO III-IV	-	-
Kroh et al [26]	Glioma WHO II-IV	-	-
Liu et al [22]	Glioma WHO III-IV	APTmean*	-
Ma et al [27]	Glioma WHO I-IV	APTmean*	2.42%
		APTmax*	2.54%
Mehrabian et al [29]	Brain metastases	APTmean	1%
Mehrabian et al [28]	Brain metastases	APTmean	1.4%
Paprottka et al [17]	Glioma WHO I-IV	-	1.79%
Park et al [23]	Glioma WHO III-IV	APT90*	1.90%/1.98%
Park et al [30]	Glioma WHO IV	APT90*	2.88%
Park et al [31]	Glioma WHO II-IV	APT90*	1.79%/1.96%
		APTmax*	2.03%
Park et al [24]	Glioma WHO II-IV	APTmean*	2.11%

Histological grading in the Roman numeric system due to the use of outdated classification systems applying

*APT90* 90% histogram value APT, *APTmax* max histogram value of APT, *APTmean* mean histogram value of APT, *WHO I-IV* World Health Organization Central Nervous System tumour grading

* Significant (*p* < 0.05) difference between signal in progression and therapy-related change

In comparative studies, multi-parametric MRI imaging that included APT imaging outperformed APT-only imaging [19–22, 24]. In contrast, APT-only imaging had higher AUCs than PWI [19, 20, 23, 24], diffusion-weighted imaging (DWI) [19, 24], diffusion tensor imaging (DTI) [24], conventional MRI [21], magnetic resonance spectroscopy (MRS) [22], and PET [31]. One study found that ASL had a higher AUC than APT [22]. Adding conventional/advanced MRI techniques to APT improved diagnostic performance in all comparative studies.

### Risk of bias and applicability

**Fig. 2:**
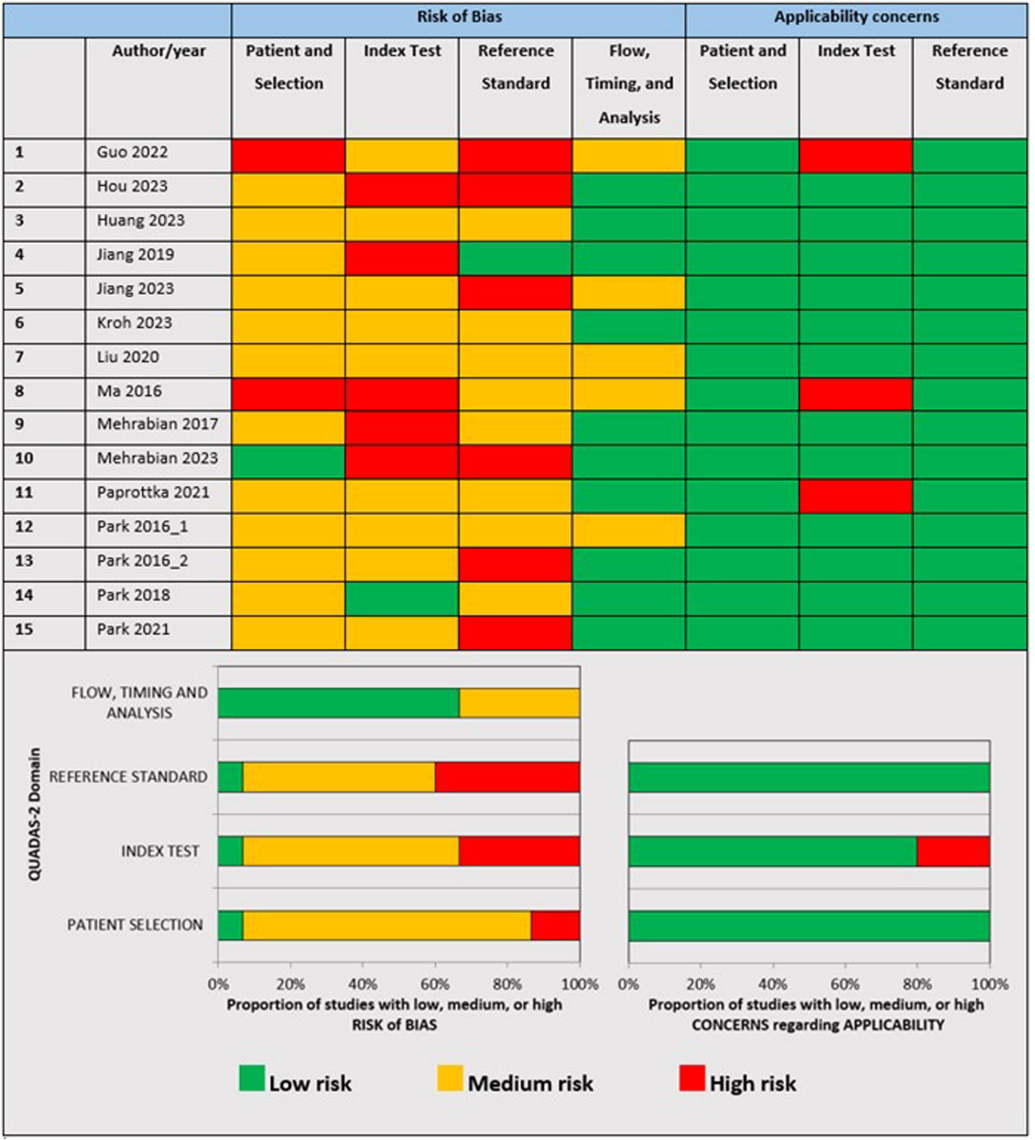
Overview of risk of bias/applicability assessment according to modified Quality Assessment of Diagnostic Accuracy Studies-2 criteria

Overall risk of bias scores were medium (*n* = 13) or high (*n* = 2) (see Fig. 2). Only three studies [24, 28, 29] mentioned the administered radiation doses relevant to reduce the risk of bias. No studies used (or mentioned the use of) the World Health Organization 2021 central nervous system tumour classification [32] for glioma grading, increasing the bias risk. Only two studies [24, 31] mentioned blinded index testing. Only one [25] study had the ideally wanted histopathological confirmation as a reference standard for all patients. Only one study [17] used a predefined threshold value. Three studies [17, 18, 27] had applicability concerns regarding the index test.

### Meta-analysis

Five hundred patients across twelve studies were included for meta-analysis, which assessed APT-only imaging (*n* = 11), multi-parametric MRI including APT (*n* = 6), PWI (*n* = 6), DWI (*n* = 2), DTI (*n* = 1) and conventional MRI (*n* = 2).

**Fig. 3:**
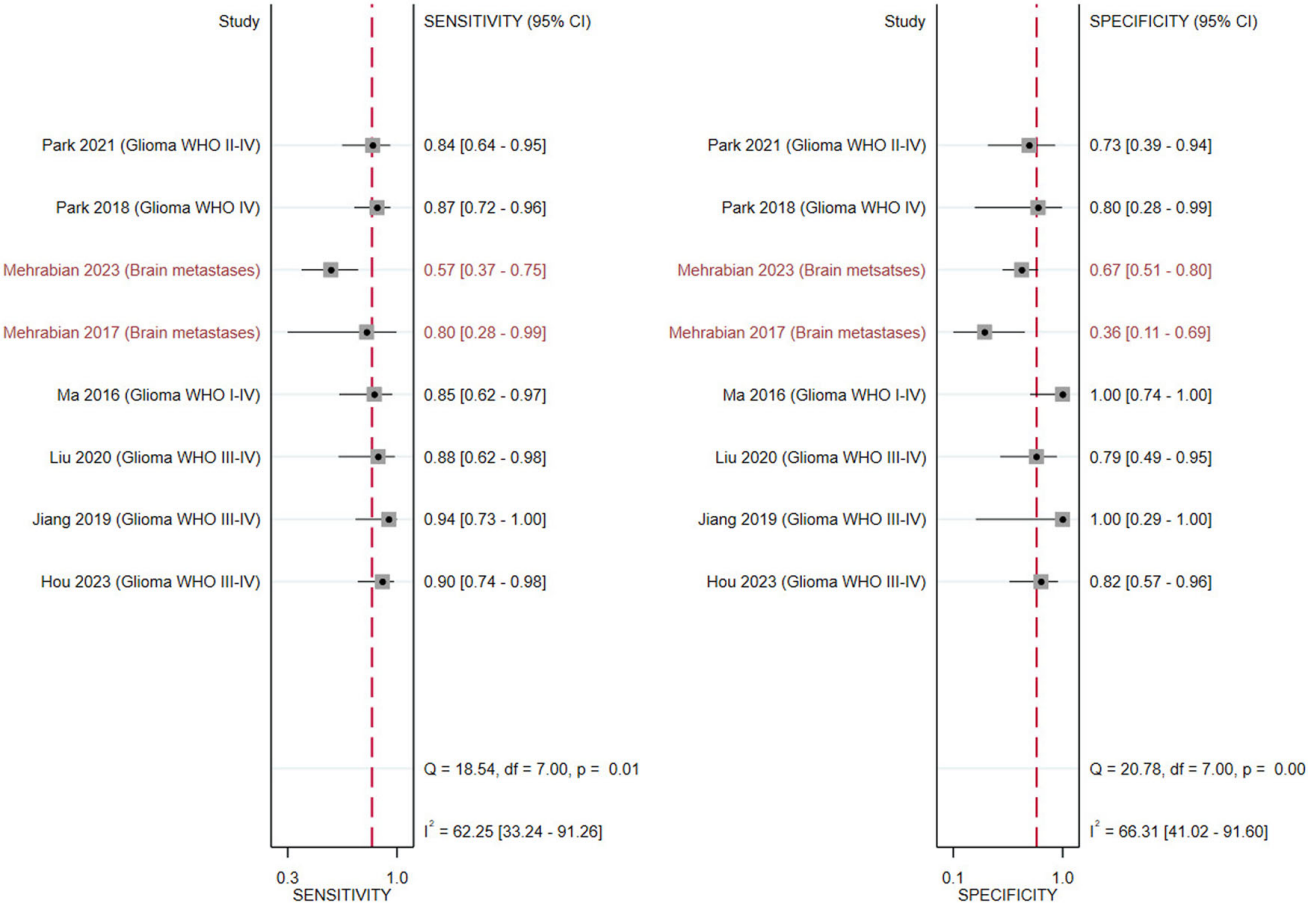
Forest plot of the diagnostic performance of amide proton transfer imaging alone for distinguishing tumour progression and recurrence from therapy-related changes in gliomas (black) and brain metastases (maroon), showing substantial heterogeneity between sensitivities (left) and specificities (right)

**Fig. 4:**
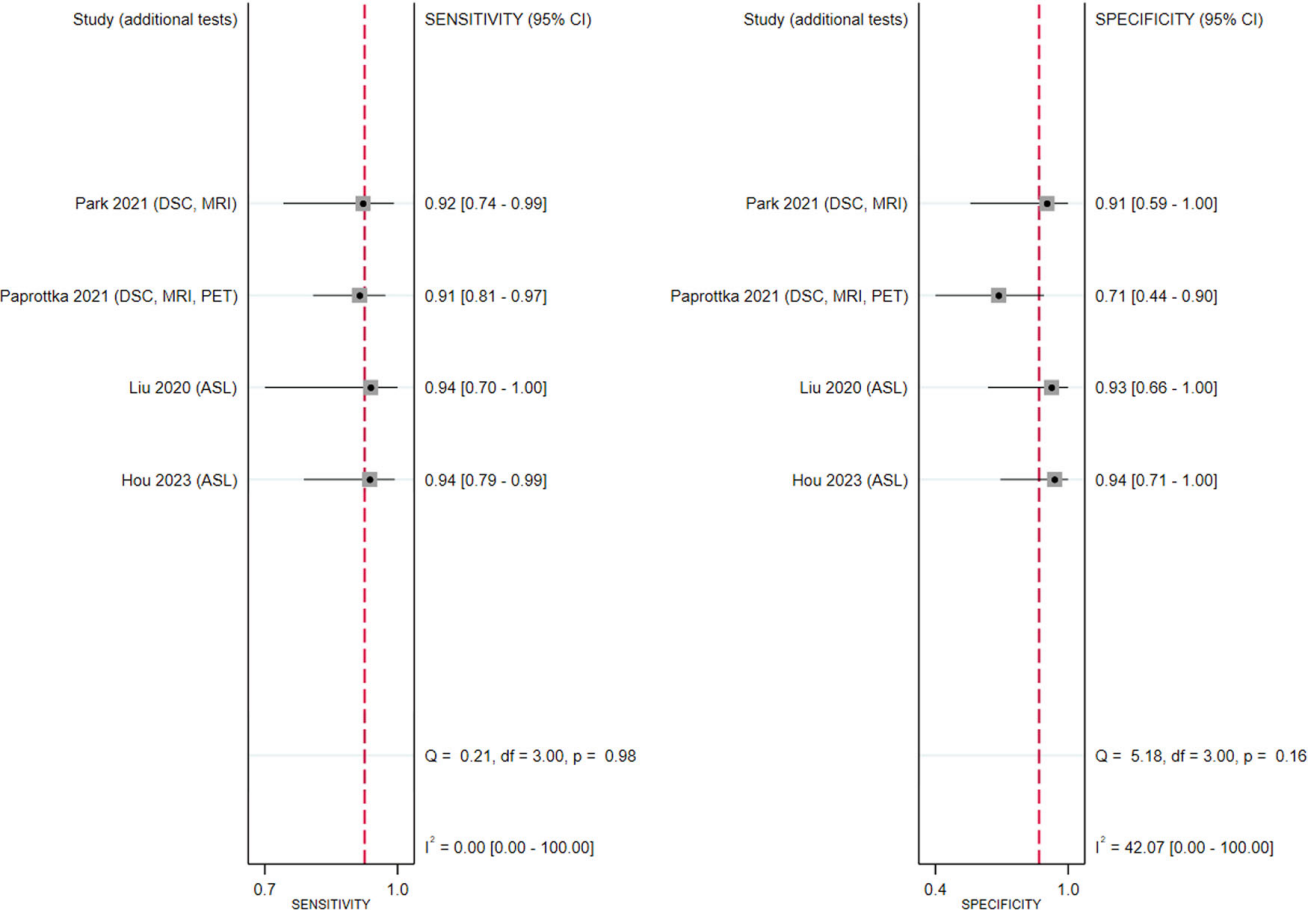
Forest plot of the diagnostic performance of amide proton transfer imaging in combination with arterial spin labelling (ASL) or dynamic susceptibility contrast (DSC) imaging and/or other imaging techniques for distinguishing tumour progression and recurrence from therapy-related changes showing mild heterogeneity between specificities (left)

**Fig. 5:**
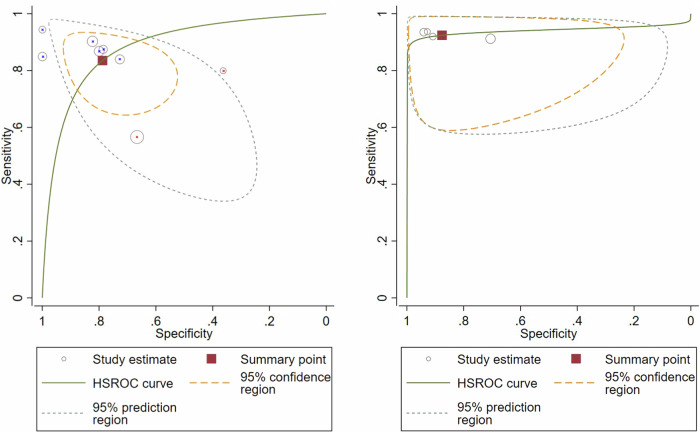
Hierarchical summary receiver operating characteristics (HSROC) curves of the diagnostic performance of amide proton transfer imaging alone (left) in gliomas (blue x) and brain metastases (red x), and in combination with other magnetic resonance imaging techniques (right) in gliomas for distinguishing tumour progression and recurrence from therapy-related changes. Summary area under the curve estimate was 0.88 [0.85–0.91] (left) and 0.94 [0.92–0.96] (right)

Sensitivity and specificity of APT alone in all tumours (*n* = 8) showed substantial inter-study heterogeneity (*I*^2^ = 62.25%; *p* < 0.01 and *I*^2^ = 66.31%; *p* < 0.001), while multi-parametric MRI including APT (*n* = 4) showed mild heterogeneity in specificity (*I*^2^ = 42.07%; *p* = 0.15) (see Figs. 3, 4). Univariate meta-regression and subgroup analysis of APT-CEST-only imaging (*n* = 8) revealed tumour type (*p* < 0.01), offset approach (*p* < 0.05), female sex percentage (*p* < 0.01) and TR prevalence (*p* < 0.01) as significant heterogeneity sources. See Fig. 5 for SROC plots of APT.

Subgroup analysis based on tumour type for APT-CEST-only, which eliminated heterogeneity, revealed significantly higher sensitivity (*p* < 0.001) and specificity (*p* = 0.015) in gliomas (0.88 [0.82–0.92]; 0.84 [0.72–0.91] respectively) compared to BM (0.64 [0.38–0.84]; 0.56 [0.33–0.77] respectively) (see Table 2).

**Table 2:** Diagnostic performance of various imaging modalities in differentiating tumour progression and recurrence from therapy-related changes in all studies and comparative studies

All studies (*N* = 9)			
	Multi-parametric MRI + APT (gliomas)	APT (gliomas)	APT (BM)
Sensitivity	0.92 [0.86–0.96]	0.88 [0.82–0.92]	0.64 [0.38–0.84]
Specificity	0.88 [0.72–0.95]	0.84 [0.72–0.91]	0.56 [0.33–0.77]
AUSROC	0.94 [0.92–0.96]	0.93 [0.90–0.95]	-
*N*	4	6	2

Comparative studies (*N* = 3)			
	APT (gliomas)	Multi-parametric MRI + APT (gliomas)	PWI (gliomas)
Sensitivity	0.87 [0.78–0.93]	0.93 [0.84–0.97]	0.65 [0.54–0.75]
Specificity	0.79 [0.64–0.88]	0.93 [0.80–0.98]	0.85 [0.68–0.94]
AUSROC	-	-	-
*N*	3	3	3

Pooled C-statistic (*N* = 7)			
	Multi-parametric MRI + APT (gliomas)	APT (gliomas)	
AUC	0.93 [0.62–0.99]	0.86 [0.60–0.96]	
*N*	4	6	

AUSROC estimate was not derivable if the number of studies was less than four

*APT* amide proton transfer, *AUC* area under the curve, *AUSROC* area under the summary receiver operator characteristics curve, *BM* brain metastases, *C-statistic* concordance statistic (equivalent to the area under the receiver operator characteristics curve), *DWI* diffusion-weighted imaging, *N* number of studies, *PWI* perfusion-weighted imaging

Three comparative studies of APT alone and multi-parametric MRI including APT-CEST [19, 22, 24] showed a mild difference (*p* = 0.056) between specificity. Three comparative studies [19, 22, 24] found a significantly higher (*p* < 0.01) APT sensitivity compared to PWI, and two [19, 24] found a significantly higher sensitivity (*p* = 0.02) for APT compared to DWI.

## Discussion

This review identified substantial potential for APT-CEST to improve the non-invasive discrimination of TRC from TP/TR in gliomas after CRT. Moreover, based on the same-population analysis, this study delivers limited evidence that APT-CEST performs similarly to PWI and better than conventional MRI, DWI, DTI and MRS for this clinical question. Combining APT-CEST with other imaging techniques in gliomas can improve diagnostic accuracy in a potential clinical setting. For BM, the level of evidence is substantially weaker due to limited data and less unequivocal outcomes.

**Fig. 6:**
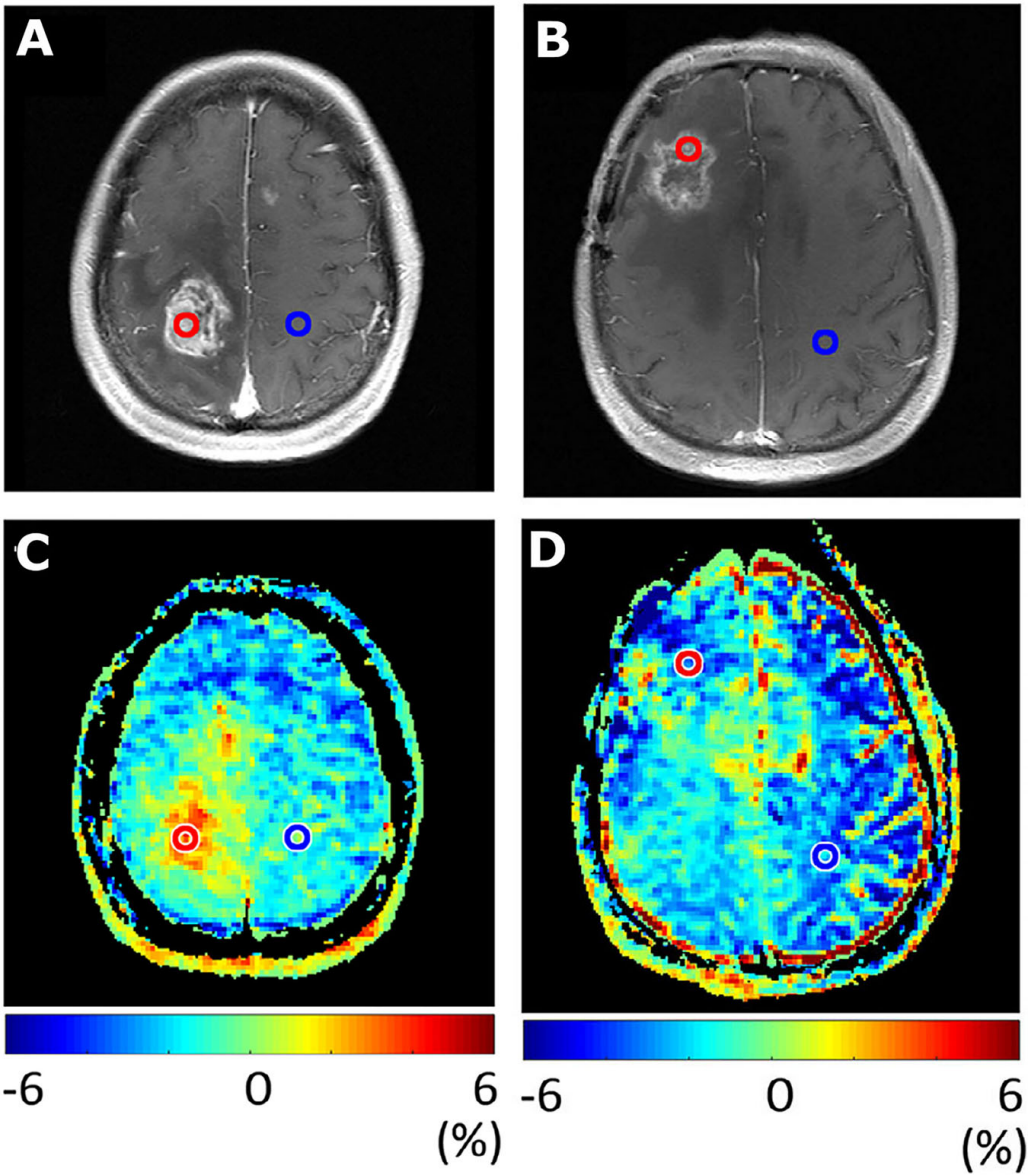
Images illustrating APT-weighted imaging (MTRasym at 3.5 ppm) in two patients after treatment with radiotherapy. Contrast enhancement on T1-weighted images was seen in patient 1, 62 months after radiotherapy treatment and resection for grade 2 astrocytoma (**A**). The additional increased MTRasym in the same patient (**C**) illustrates tumour recurrence, which was confirmed as grade 4 glioblastoma after repeat surgery. Contrast enhancement on T1-weighted images was seen in patient 2, 14 months after chemoradiotherapy of grade 3 astrocytoma, with regional anaplastic oligodendroglioma (**B**). The additional MTRasym in patient 2 (**D**) illustrates low values, indicating treatment effect, which was confirmed as radiation necrosis with histopathology after repeat surgery. Image reproduced via open access CC license and permission from ref. [11]

The findings of this study corroborate preliminary findings from an earlier review of gliomas [12]. The theoretical backdrop is that the APT signal is relatively higher in TP/TR due to increased cellularity and protein content in tumour tissue compared to RT-induced coagulative necrosis and loss of cytosolic components in TRC. As intracellular cytosolic proteins are a potential source of APT signal, loss of these components could lead to decreased saturation transfer from amide to water and subsequent signal decrease. APT signal increase in large cysts or haemorrhage may limit accuracy but can be corrected through co-registration on conventional MRI [19], further increasing diagnostic performance [21] (see Fig. 6). Moreover, APT combined with PWI allows for assessing two distinct tumour characteristics, protein content and neovascularisation, which could work synergistically and correct their individual limitations to provide higher diagnostic performance. However, this review did not identify a discriminatory capability in BM using MTR_asym(3.5 ppm)._ In contrast, PWI, PET, and conventional MRI all demonstrated some capability to differentiate TP/TR from TRC in BM [33]. Peculiarly, both BM APT-CEST articles included the highest TRC percentage among all studies, which is attributable to stereotactic radiosurgery in BM, causing a high incidence of RN. This could explain why MTR_asym(3.5 ppm)_ imaging performed poorly compared to glioma. The authors postulate that the subtraction of MTR_Amide_ from MTR_NOE_, both of which did individually provide significant discriminatory capacities, could diminish the signal difference between TP/TR and TRC. One metastasis study [29] also exclusively used a non-standard saturation pulse amplitude (0.52 μT), resulting in a large NOE contribution and low MTR_asym(3.5 ppm)_ signal [34]. Another explanation for the lack of signal difference in BM could include an a priori lower signal intensity in metastases compared to primary brain tumours [35]. Furthermore, both studies included various types of BM, possibly confounding APT signals as they differ in histological characteristics. Unfortunately, literature on the APT signal in different BM types is lacking. The underrepresentation of BM in this study, and APT-CEST research in general, makes drawing any conclusions about the clinical applicability challenging. Additional research is required, using various acquisition parameters and processing metrics, to determine the utility of APT-CEST in treatment response assessment in metastasis patients treated with stereotactic radiosurgery in a clinical setting. Kroh et al [26] was the only glioma study where APT performed insufficiently. Z-spectral fitting-based and relaxation-compensated approaches did not provide significant diagnostic performance either. The latter phenomenon can be explained through a large saturation bandwidth at 2 μT amplitude at 3 T, which can make APT signal invisible [10]. NOE, direct effect, and amide signals all contribute, in varying degrees, to the APT-weighted signal. The amount of APT-weighted signal in TP/TR that is a result of pure amide proton transfer, is a matter yet to be elucidated. In addition, there are T1-contributions and spill-over effects, the latter of which could be corrected for through a relaxation-compensated approach. More research is required to validate the utility of Z-spectral fitting-based and relaxation-compensated approaches in APT imaging in post-treatment response assessment.

Some limitations apply to the articles included in this review and meta-analysis. First, the effect of irradiation dose, which possibly increases RN incidence, on APT signal could not be investigated due to unreported doses in most studies. Additionally, methylguanine-DNA methyltransferase (MGMT) methylation might influence the incidence of PsP in glioblastomas [36]. Unfortunately, the included studies either did not report or separately analyse different tumour mutations. Additionally, the fact that most studies did not exclusively apply histopathological verification as a reference standard, and used outdated WHO criteria for glioma grading raises concerns regarding the clinical applicability of these results. Furthermore, some studies assessed both lower and high-grade glioma (LGG/HGG), possibly confounding APT signal as recent literature suggests that APT-weighted signal differs between LGG and HGG [37]. However, glioma subtypes did not appear to cause significant heterogeneity in this meta-analysis, which could result from the small number of patients with LGG compared to HGG. To account for threshold effect-induced heterogeneity, this review provides an overview of the diagnostic performance of APT-CEST at various imaging parameters with their respective optimal thresholds. Future research can focus on validating and/or implementing these parameters and thresholds to further explore and facilitate clinical implementation. Additionally, this review has several limitations. First, this review and meta-analysis only includes a small number of studies because of the novelty of and the limited literature regarding this subject. Taking the limited data and significant inter-study heterogeneity at the outcome level into account, the pooled forest plot results were omitted. While the meta-regression and subgroup analysis may hint at possible biases and/or heterogeneity-inducing factors in the data, the interpretation of these results is challenging considering the low sample sizes and low statistical power resulting from a limited number of studies. Another limitation is that the included studies assessed patients within 2 years after radiotherapy completion to include both PsP and RN, which both classify as TRC. Unfortunately, this leads to a large variation in the interval between therapy and APT imaging across studies. Furthermore, some studies included outliers, with patients receiving APT imaging up to 10 years [21] after completion of radiotherapy. Moreover, the gold standard for differentiation between TP/TR and TRC is histopathological assessment after biopsy or resection. It was thus regarded as the optimal reference standard in this review, but it is still prone to sampling bias [38]. Furthermore, two studies [18, 21] had (partially) overlapping cohorts with two other studies [25, 27]. However, both studies were excluded from the meta-analysis to avoid cohort overlap. The number of included studies comparing APT-CEST to other imaging techniques is small. For this reason, pooled results of comparative studies were reported separately. The forest plot analysis, however, provides a visual indicator for future comparable studies regarding the potentially achievable sensitivities and specificities, which for glioma range close to those of other quantitative MRI techniques, hinting at the clinical utility of APT-CEST [11]. Additionally, some aspects need to be noted in this review. Although Z-spectral fitting-based and relaxation-compensated approaches did perform well in BM [28, 29], only results of MTR_asym_(3.5 ppm) APT at 3 T were included in the meta-analysis to facilitate the harmonisation of efforts in future research, as MTR_asym_(3.5 ppm) at 3 T is most commonly used for brain tumour assessment in a clinical setting [10]. While the MRI parameters for APT image acquisition varied, they did not cause any statistical heterogeneity after the removal of BM studies. Furthermore, the risk of bias assessment in this review was quite strict to better represent the real-world applicability of APT-CEST for treatment response assessment. Finally, publication bias was not assessed in this review due to the limited number of studies included for analysis. Additionally, funnel plots of the diagnostic odds ratio bode low statistical power and are difficult to interpret if multiple index tests are being evaluated [39].

In conclusion, APT-CEST has shown significant promise in discriminating TP/TR from TRC in post-treatment gliomas. However, this has not been demonstrated in metastases. In addition, APT-CEST imaging has shown limited evidence for superior diagnostic performance to other conventional or advanced MRI techniques. A combination of APT-CEST with PWI and/or conventional MRI techniques appears to provide the best diagnostic performance, outperforming each single imaging modality. As such, APT-CEST has demonstrated potential as an alternative to biopsy in post-treatment response assessment of gliomas in a clinical setting. Further validation of these results, as well as a higher degree of inter-centre technique harmonisation, are warranted for future research.

## Supplementary information

ELECTRONIC SUPPLEMENTARY MATERIAL

## Abbreviations

APT: Amide proton transfer
BM: Brain metastasis
CEST: Chemical exchange saturation transfer
CRT: Chemoradiotherapy
HGG: High-grade glioma
LGG: Lower-grade glioma
MTR_Amide_: Magnetisation transfer ratio of amide (+3.5 parts per million)
MTR_asym_(3.5 ppm): Magnetisation transfer ratio asymmetry analysis at ± 3.5 parts per million
MTR_NOE_: Magnetisation transfer ratio of nuclear Overhauser effect (−3.5 parts per million)
PsP: Pseudoprogression
RN: Radiation necrosis
RT: Radiotherapy
TP: Tumour progression
TR: Tumour recurrence
TRC: Therapy-related changes
