# Supplementary material for: Performance of amide proton transfer imaging to differentiate true progression from therapy-related changes in gliomas and metastases
Source: Eur Radiol. 2024 Aug 12;35(2):580–91. doi: 10.1007/s00330-024-11004-y (PMC11782315; doi:10.1007/s00330-024-11004-y)
Supplement: Supplementary file 1 — ELECTRONIC SUPPLEMENTARY MATERIAL [file 330_2024_11004_MOESM1_ESM.pdf]

Performance of Amide Proton Transfer Imaging to Differentiate True Progression from Therapy-related Changes in Gliomas and Metastases

ELECTRONIC SUPPLEMENTARY MATERIAL

Search string

Aspects:

1. tumour (gliomas and metastases)
2. Imaging (APT-CEST)
3. Treatment response (Therapy-related changes and true progression/recurrence)

Notes:

- Some characters like '/' are not applicable to some databases like Cochrane Library. These were excluded.
- **AND** Boolean operator was included in aspect 3 (therapy-response assessment) between terms relating to therapy-related changes and terms relating to true progression/recurrence. This was to only search for records that study both phenomena as they need to be differentiated from one another.
- Due to uncertainty among reviewers regarding the inclusion of a hyphen (-) in the database searches, both variants (with and without a hyphen) were included.
- 'Therapy response' and its variations were intentionally excluded from aspect 3 of the search as they include complete response, partial response, and stable disease as well.

Pubmed:

((((therapy-related change*[Title/Abstract] OR therapy related change*[Title/Abstract] OR pseudoprogression[Title/Abstract] OR radiation necrosis[Title/Abstract] OR radionecrosis[Title/Abstract] OR RN[Title/Abstract] OR radiation-induced necrosis[Title/Abstract] OR radiation induced necrosis[Title/Abstract] OR necrosis[Title/Abstract] OR PsP[Title/Abstract] OR TRC[Title/Abstract] OR treatment-related change*[Title/Abstract] OR treatment related change*[Title/Abstract] OR therapy change*[Title/Abstract] OR therapy effect*[Title/Abstract] OR treatment effect*[Title/Abstract] OR treatment-related effect*[Title/Abstract] OR treatment related effect*[Title/Abstract] OR treatment-induced effect*[Title/Abstract] OR treatment induced effect*[Title/Abstract] OR treatment-induced change*[Title/Abstract] OR treatment induced change*[Title/Abstract] OR therapy-induced change*[Title/Abstract] OR therapy induced change*[Title/Abstract] OR therapy-induced effect*[Title/Abstract] OR therapy induced effect*[Title/Abstract] OR therapy-related effect*[Title/Abstract] OR therapy related effect*[Title/Abstract] OR therapy-induced necrosis[Title/Abstract] OR therapy induced necrosis[Title/Abstract] OR treatment-induced necrosis[Title/Abstract] OR treatment induced necrosis[Title/Abstract] OR TE[Title/Abstract] OR TrE[Title/Abstract] OR treatment-related inflammation[Title/Abstract] OR treatment related inflammation[Title/Abstract] OR therapy-

related inflammation[Title/Abstract] OR therapy related inflammation[Title/Abstract] OR treatment-related necrosis[Title/Abstract] OR treatment related necrosis[Title/Abstract] OR therapy-related necrosis[Title/Abstract] OR therapy related necrosis[Title/Abstract] OR post-radiotherapy change*[Title/Abstract] OR post radiotherapy change*[Title/Abstract] OR post-radiotherapy necrosis[Title/Abstract] OR post radiotherapy necrosis[Title/Abstract] OR post-radiotherapy inflammation[Title/Abstract] OR post radiotherapy inflammation[Title/Abstract]) OR (Abnormalities, radiation induced[MeSH Terms])) AND ((progression[Title/Abstract] OR true progression[Title/Abstract] OR real progression[Title/Abstract] OR disease progression[Title/Abstract] OR progressive disease[Title/Abstract] OR tumour progression[Title/Abstract] OR tumour progression[Title/Abstract] OR progressive tumour*[Title/Abstract] OR progressive tumour*[Title/Abstract] OR recurrence*[Title/Abstract] OR tumour recurrence*[Title/Abstract] OR tumour recurrence*[Title/Abstract] OR recurrent tumour*[Title/Abstract] OR recurrent tumour*[Title/Abstract] OR recurrent disease*[Title/Abstract] OR true tumour progression[Title/Abstract] OR true tumour progression[Title/Abstract] OR true tumour recurrence*[Title/Abstract] OR true tumour recurrence*[Title/Abstract] OR TTP[Title/Abstract] OR TP[Title/Abstract] OR TR[Title/Abstract] OR recurrent glioma*[Title/Abstract] OR recurrent metastasis[Title/Abstract] OR recurrent metastases[Title/Abstract] OR recurrent astrocytoma*[Title/Abstract] OR progressive glioma*[Title/Abstract] OR progressive astrocytoma*[Title/Abstract] OR progressive metastasis[Title/Abstract] OR progressive metastases[Title/Abstract] OR progressive glioblastoma[Title/Abstract] OR progressive GBM[Title/Abstract] OR recurrent glioblastoma[Title/Abstract] OR recurrent GBM[Title/Abstract] OR real tumour progression[Title/Abstract] OR real tumour progression[Title/Abstract] OR real tumour recurrence*[Title/Abstract] OR real tumour recurrence*[Title/Abstract] OR true progressive tumour*[Title/Abstract] OR true progressive tumour*[Title/Abstract] OR true recurrent tumour*[Title/Abstract] OR true recurrent tumour*[Title/Abstract] OR TuR[Title/Abstract]) OR (disease progression[MeSH Terms]))) AND (amide proton transfer[Title/Abstract] OR amine proton transfer[Title/Abstract] OR amide proton transfer weighted[Title/Abstract] OR amine proton transfer weighted[Title/Abstract] OR APT[Title/Abstract] OR CEST[Title/Abstract] OR APTw[Title/Abstract] OR chemical exchange saturation transfer[Title/Abstract] OR APT-CEST[Title/Abstract] OR APT/CEST[Title/Abstract] OR amide proton transfer imaging[Title/Abstract] OR amine proton transfer imaging[Title/Abstract] OR CEST imaging[Title/Abstract] OR APT imaging[Title/Abstract] OR amide proton transfer weighted imaging[Title/Abstract] OR amine proton transfer weighted imaging[Title/Abstract] OR chemical exchange saturation transfer imaging[Title/Abstract] OR APT-*[Title/Abstract] OR CEST-*[Title/Abstract])) AND (((((((glioma*[Title/Abstract] OR glioblastoma*[Title/Abstract] OR GBM[Title/Abstract] OR astrocytoma*[Title/Abstract] OR anaplastic astrocytoma*[Title/Abstract] OR high-grade glioma*[Title/Abstract] OR high grade glioma*[Title/Abstract] OR low-grade glioma*[Title/Abstract] OR low grade glioma*[Title/Abstract] OR high-grade astrocytoma*[Title/Abstract] OR high grade astrocytoma*[Title/Abstract] OR low-grade astrocytoma*[Title/Abstract] OR low grade astrocytoma*[Title/Abstract] OR high-grade tumour*[Title/Abstract] OR high grade tumour*[Title/Abstract] OR low-grade tumour*[Title/Abstract] OR low grade tumour*[Title/Abstract] OR high-grade tumour*[Title/Abstract] OR high grade tumour*[Title/Abstract] OR low-grade tumour*[Title/Abstract] OR low grade tumour*[Title/Abstract] OR brain cancer*[Title/Abstract] OR brain tumour*[Title/Abstract] OR brain tumour*[Title/Abstract] OR brain metastasis[Title/Abstract] OR brain metastases[Title/Abstract] OR cerebral

metastasis[Title/Abstract] OR cerebral metastases[Title/Abstract] OR intra-axial brain tumour*[Title/Abstract] OR intra-axial brain tumour*[Title/Abstract] OR intra axial brain tumour*[Title/Abstract] OR intra axial brain tumour*[Title/Abstract] OR intra-axial tumour*[Title/Abstract] OR intra-axial tumour*[Title/Abstract] OR intra axial tumour*[Title/Abstract] OR intra axial tumour*[Title/Abstract] OR intra-axial metastasis[Title/Abstract] OR intra-axial metastases[Title/Abstract] OR LGG[Title/Abstract] OR HGG[Title/Abstract] OR intra axial metastasis[Title/Abstract] OR intra axial metastases[Title/Abstract] OR intra-axial brain metastasis[Title/Abstract] OR intra-axial brain metastases[Title/Abstract] OR intra axial brain metastasis[Title/Abstract] OR intra axial brain metastases[Title/Abstract] OR diffuse glioma*[Title/Abstract] OR diffuse astrocytoma*[Title/Abstract] OR oligoastrocytoma*[Title/Abstract] OR oligodendroglioma*[Title/Abstract] OR cerebral tumour*[Title/Abstract] OR cerebral tumour*[Title/Abstract] OR metastasis[Title/Abstract] OR metastases[Title/Abstract] OR CNS neoplasm*[Title/Abstract] OR neoplasm*[Title/Abstract] OR brain neoplasm*[Title/Abstract] OR cerebral neoplasm*[Title/Abstract] OR cerebral malignancy[Title/Abstract] OR cerebral malignancies[Title/Abstract] OR brain malignancy[Title/Abstract] OR brain malignancies[Title/Abstract]) OR (glioma[MeSH Terms])) OR (anaplastic astrocytoma[MeSH Terms])) OR (anaplastic astrocytomas[MeSH Terms])) OR (astrocytoma[MeSH Terms])) OR (astrocytomas[MeSH Terms])) OR (metastases[MeSH Terms]))

Web of Science:

TS=(therapy-related change* OR therapy related change* OR pseudoprogression OR radiation necrosis OR radionecrosis OR RN OR radiation-induced necrosis OR radiation induced necrosis OR necrosis OR PsP OR TRC OR treatment-related change* OR treatment related change* OR therapy change* OR therapy effect* OR treatment effect* OR treatment-related effect* OR treatment related effect* OR treatment-induced effect* OR treatment induced effect* OR treatment-induced change* OR treatment induced change* OR therapy-induced change* OR therapy induced change* OR therapy-induced effect* OR therapy induced effect* OR therapy-related effect* OR therapy related effect* OR therapy-induced necrosis OR therapy induced necrosis OR treatment-induced necrosis OR treatment induced necrosis OR TE OR TrE OR treatment-related inflammation OR treatment related inflammation OR therapy-related inflammation OR therapy related inflammation OR treatment-related necrosis OR treatment related necrosis OR therapy-related necrosis OR therapy related necrosis OR post-radiotherapy change* OR post radiotherapy change* OR post-radiotherapy necrosis OR post radiotherapy necrosis OR post-radiotherapy inflammation OR post radiotherapy inflammation) AND TS=(progression OR true progression OR real progression OR disease progression OR progressive disease OR tumour progression OR tumour progression OR progressive tumour* OR progressive tumour* OR recurrence* OR tumour recurrence* OR tumour recurrence* OR recurrent tumour* OR recurrent tumour* OR recurrent disease* OR true tumour progression OR true tumour progression OR true tumour recurrence* OR true tumour recurrence* OR TTP OR TP OR TR OR recurrent glioma* OR recurrent metastasis OR recurrent metastases OR recurrent astrocytoma* OR progressive glioma* OR progressive astrocytoma* OR progressive metastasis OR progressive metastases OR progressive glioblastoma OR progressive GBM OR recurrent glioblastoma OR recurrent GBM OR real tumour progression or real tumour progression OR real tumour recurrence* OR real tumour recurrence* OR true progressive tumour* OR true progressive tumour* OR true recurrent tumour* OR true recurrent tumour* OR TuR) AND TS=(amide proton transfer OR amine proton transfer OR amide proton transfer

weighted OR amine proton transfer weighted OR APT OR CEST OR APTw OR chemical exchange saturation transfer OR APT-CEST OR APT/CEST OR amide proton transfer imaging OR amine proton transfer imaging OR CEST imaging OR APT imaging OR amide proton transfer weighted imaging OR amine proton transfer weighted imaging OR chemical exchange saturation transfer imaging OR APT-* OR CEST-* OR APT* OR CEST*) AND TS=(glioma* OR glioblastoma* OR GBM OR astrocytoma* OR anaplastic astrocytoma* OR high-grade glioma* OR high grade glioma* OR low-grade glioma* OR low grade glioma* OR high-grade astrocytoma* OR high grade astrocytoma* OR low-grade astrocytoma* OR low grade astrocytoma* OR high-grade tumour* OR high grade tumour* OR low-grade tumour* OR low grade tumour* OR high-grade tumour* OR high grade tumour* OR low-grade tumour* OR low grade tumour* OR brain cancer* OR brain tumour* OR brain tumour* OR brain metastasis OR brain metastases OR cerebral metastasis OR cerebral metastases OR intra-axial brain tumour* OR intra-axial brain tumour* OR intra axial brain tumour* OR intra axial brain tumour* OR intra-axial tumour* OR intra-axial tumour* OR intra axial tumour* OR intra axial tumour* OR intra-axial metastasis OR intra-axial metastases OR intra axial metastasis OR intra axial metastases OR intra-axial brain metastasis OR intra-axial brain metastases OR intra axial brain metastasis OR intra axial brain metastases OR diffuse glioma* OR diffuse astrocytoma* OR oligoastrocytoma* OR oligodendroglioma* OR cerebral tumour* OR cerebral tumour* OR metastasis OR metastases OR CNS neoplasm* OR neoplasm* OR brain neoplasm* OR cerebral neoplasm* OR cerebral malignancy OR cerebral malignancies OR brain malignancy OR brain malignancies OR LGG OR HGG)

EMBASE:

('therapy-related change*':ti,ab,kw OR 'therapy related change*':ti,ab,kw OR pseudoprogression:ti,ab,kw OR 'radiation necrosis':ti,ab,kw OR radionecrosis:ti,ab,kw OR rn:ti,ab,kw OR 'radiation-induced necrosis':ti,ab,kw OR 'radiation induced necrosis':ti,ab,kw OR necrosis:ti,ab,kw OR psp:ti,ab,kw OR trc:ti,ab,kw OR 'treatment-related change*':ti,ab,kw OR 'treatment related change*':ti,ab,kw OR 'therapy change*':ti,ab,kw OR 'therapy effect*':ti,ab,kw OR 'treatment effect*':ti,ab,kw OR 'treatment-related effect*':ti,ab,kw OR 'treatment related effect*':ti,ab,kw OR 'treatment-induced effect*':ti,ab,kw OR 'treatment induced effect*':ti,ab,kw OR 'treatment-induced change*':ti,ab,kw OR 'treatment induced change*':ti,ab,kw OR 'therapy-induced change*':ti,ab,kw OR 'therapy induced change*':ti,ab,kw OR 'therapy-induced effect*':ti,ab,kw OR 'therapy induced effect*':ti,ab,kw OR 'therapy-related effect*':ti,ab,kw OR 'therapy related effect*':ti,ab,kw OR 'therapy-induced necrosis':ti,ab,kw OR 'therapy induced necrosis':ti,ab,kw OR 'treatment-induced necrosis':ti,ab,kw OR 'treatment induced necrosis':ti,ab,kw OR te:ti,ab,kw OR tre:ti,ab,kw OR 'treatment-related inflammation':ti,ab,kw OR 'treatment related inflammation':ti,ab,kw OR 'therapy-related inflammation':ti,ab,kw OR 'therapy related inflammation':ti,ab,kw OR 'treatment-related necrosis':ti,ab,kw OR 'treatment related necrosis':ti,ab,kw OR 'therapy-related necrosis':ti,ab,kw OR 'therapy related necrosis':ti,ab,kw OR 'post-radiotherapy change*':ti,ab,kw OR 'post radiotherapy change*':ti,ab,kw OR 'post-radiotherapy necrosis':ti,ab,kw OR 'post radiotherapy necrosis':ti,ab,kw OR 'post-radiotherapy inflammation':ti,ab,kw OR 'post radiotherapy inflammation':ti,ab,kw) AND (progression:ti,ab,kw OR 'true progression':ti,ab,kw OR 'real progression':ti,ab,kw OR 'disease progression':ti,ab,kw OR 'progressive disease':ti,ab,kw OR 'tumour progression':ti,ab,kw OR 'tumour progression':ti,ab,kw OR 'progressive tumour*':ti,ab,kw OR 'progressive tumour*':ti,ab,kw OR

recurrence*:ti,ab,kw OR 'tumour recurrence*':ti,ab,kw OR 'tumour recurrence*':ti,ab,kw OR 'recurrent tumour*':ti,ab,kw OR 'recurrent tumour*':ti,ab,kw OR 'recurrent disease*':ti,ab,kw OR 'true tumour progression':ti,ab,kw OR 'true tumour progression':ti,ab,kw OR 'true tumour recurrence*':ti,ab,kw OR 'true tumour recurrence*':ti,ab,kw OR ttp:ti,ab,kw OR tp:ti,ab,kw OR tr:ti,ab,kw OR 'recurrent glioma*':ti,ab,kw OR 'recurrent metastasis':ti,ab,kw OR 'recurrent metastases':ti,ab,kw OR 'recurrent astrocytoma*':ti,ab,kw OR 'progressive glioma*':ti,ab,kw OR 'progressive astrocytoma*':ti,ab,kw OR 'progressive metastasis':ti,ab,kw OR 'progressive metastases':ti,ab,kw OR 'progressive glioblastoma':ti,ab,kw OR 'progressive gbm':ti,ab,kw OR 'recurrent glioblastoma':ti,ab,kw OR 'recurrent gbm':ti,ab,kw OR 'real tumour progression':ti,ab,kw OR 'real tumour progression':ti,ab,kw OR 'real tumour recurrence*':ti,ab,kw OR 'real tumour recurrence*':ti,ab,kw OR 'true progressive tumour*':ti,ab,kw OR 'true progressive tumour*':ti,ab,kw OR 'true recurrent tumour*':ti,ab,kw OR 'true recurrent tumour*':ti,ab,kw OR tur:ti,ab,kw) AND ('amide proton transfer':ti,ab,kw OR 'amine proton transfer':ti,ab,kw OR 'amide proton transfer weighted':ti,ab,kw OR 'amine proton transfer weighted':ti,ab,kw OR apt:ti,ab,kw OR cest:ti,ab,kw OR aptw:ti,ab,kw OR 'chemical exchange saturation transfer':ti,ab,kw OR 'apt cest':ti,ab,kw OR 'apt/cest':ti,ab,kw OR 'amide proton transfer imaging':ti,ab,kw OR 'amine proton transfer imaging':ti,ab,kw OR 'cest imaging':ti,ab,kw OR 'apt imaging':ti,ab,kw OR 'amide proton transfer weighted imaging':ti,ab,kw OR 'amine proton transfer weighted imaging':ti,ab,kw OR 'chemical exchange saturation transfer imaging':ti,ab,kw OR apt*:ti,ab,kw OR cest*:ti,ab,kw) AND (glioma*:ti,ab,kw OR glioblastoma*:ti,ab,kw OR gbm:ti,ab,kw OR astrocytoma*:ti,ab,kw OR 'anaplastic astrocytoma*':ti,ab,kw OR 'high-grade glioma*':ti,ab,kw OR 'high grade glioma*':ti,ab,kw OR 'low-grade glioma*':ti,ab,kw OR 'low grade glioma*':ti,ab,kw OR 'high-grade astrocytoma*':ti,ab,kw OR 'high grade astrocytoma*':ti,ab,kw OR 'low-grade astrocytoma*':ti,ab,kw OR 'low grade astrocytoma*':ti,ab,kw OR 'high-grade tumour*':ti,ab,kw OR 'high grade tumour*':ti,ab,kw OR 'low-grade tumour*':ti,ab,kw OR 'low grade tumour*':ti,ab,kw OR 'high-grade tumour*':ti,ab,kw OR 'high grade tumour*':ti,ab,kw OR 'low-grade tumour*':ti,ab,kw OR 'low grade tumour*':ti,ab,kw OR 'brain cancer*':ti,ab,kw OR 'brain tumour*':ti,ab,kw OR 'brain tumour*':ti,ab,kw OR 'brain metastasis':ti,ab,kw OR 'brain metastases':ti,ab,kw OR 'cerebral metastasis':ti,ab,kw OR 'cerebral metastases':ti,ab,kw OR 'intra-axial brain tumour*':ti,ab,kw OR 'intra-axial brain tumour*':ti,ab,kw OR 'intra axial brain tumour*':ti,ab,kw OR 'intra axial brain tumour*':ti,ab,kw OR 'intra-axial tumour*':ti,ab,kw OR 'intra-axial tumour*':ti,ab,kw OR 'intra axial tumour*':ti,ab,kw OR 'intra axial tumour*':ti,ab,kw OR 'intra-axial metastasis':ti,ab,kw OR 'intra-axial metastases':ti,ab,kw OR 'intra axial metastasis':ti,ab,kw OR 'intra axial metastases':ti,ab,kw OR 'intra-axial brain metastasis':ti,ab,kw OR 'intra-axial brain metastases':ti,ab,kw OR 'intra axial brain metastasis':ti,ab,kw OR 'intra axial brain metastases':ti,ab,kw OR 'diffuse glioma*':ti,ab,kw OR 'diffuse astrocytoma*':ti,ab,kw OR oligoastrocytoma*:ti,ab,kw OR oligodendroglioma*:ti,ab,kw OR 'cerebral tumour*':ti,ab,kw OR 'cerebral tumour*':ti,ab,kw OR metastasis:ti,ab,kw OR metastases:ti,ab,kw OR 'cns neoplasm*':ti,ab,kw OR neoplasm*:ti,ab,kw OR 'brain neoplasm*':ti,ab,kw OR 'cerebral neoplasm*':ti,ab,kw OR 'cerebral malignancy':ti,ab,kw OR 'cerebral malignancies':ti,ab,kw OR 'brain malignancy':ti,ab,kw OR 'brain malignancies':ti,ab,kw OR 'LGG':ti,ab,kw OR 'HGG':ti,ab,kw)

Cochrane Library:

(therapy-related change* OR therapy related change* OR pseudoprogression OR radiation necrosis OR radionecrosis OR RN OR radiation-induced necrosis OR radiation induced necrosis OR necrosis OR PsP OR TRC OR treatment-related change* OR treatment related change* OR therapy change* OR therapy effect* OR treatment effect* OR treatment-related effect* OR treatment related effect* OR treatment-induced effect* OR treatment induced effect* OR treatment-induced change* OR treatment induced change* OR therapy-induced change* OR therapy induced change* OR therapy-induced effect* OR therapy induced effect* OR therapy-related effect* OR therapy related effect* OR therapy-induced necrosis OR therapy induced necrosis OR treatment-induced necrosis OR treatment induced necrosis OR TE OR TrE OR treatment-related inflammation OR treatment related inflammation OR therapy-related inflammation OR therapy related inflammation OR treatment-related necrosis OR treatment related necrosis OR therapy-related necrosis OR therapy related necrosis OR post-radiotherapy change* OR post radiotherapy change* OR post-radiotherapy necrosis OR post radiotherapy necrosis OR post-radiotherapy inflammation OR post radiotherapy inflammation):ti,ab,kw AND (amide proton transfer OR amine proton transfer OR amide proton transfer weighted OR amine proton transfer weighted OR APT OR CEST OR APTw OR chemical exchange saturation transfer OR APT-CEST OR amide proton transfer imaging OR amine proton transfer imaging OR CEST imaging OR APT imaging OR amide proton transfer weighted imaging OR amine proton transfer weighted imaging OR chemical exchange saturation transfer imaging):ti,ab,kw AND (progression OR true progression OR real progression OR disease progression OR progressive disease OR tumour progression OR tumour progression OR progressive tumour* OR progressive tumour* OR recurrence* OR tumour recurrence* OR tumour recurrence* OR recurrent tumour* OR recurrent tumour* OR recurrent disease* OR true tumour progression OR true tumour progression OR true tumour recurrence* OR true tumour recurrence* OR TTP OR TP OR TR OR recurrent glioma* OR recurrent metastasis OR recurrent metastases OR recurrent astrocytoma* OR progressive glioma* OR progressive astrocytoma* OR progressive metastasis OR progressive metastases OR progressive glioblastoma OR progressive GBM OR recurrent glioblastoma OR recurrent GBM OR real tumour progression or real tumour progression OR real tumour recurrence* OR real tumour recurrence* OR true progressive tumour* OR true progressive tumour* OR true recurrent tumour* OR true recurrent tumour* OR TuR):ti,ab,kw AND (glioma* OR glioblastoma* OR GBM OR astrocytoma* OR anaplastic astrocytoma* OR high-grade glioma* OR high grade glioma* OR low-grade glioma* OR low grade glioma* OR high-grade astrocytoma* OR high grade astrocytoma* OR low-grade astrocytoma* OR low grade astrocytoma* OR high-grade tumour* OR high grade tumour* OR low-grade tumour* OR low grade tumour* OR high-grade tumour* OR high grade tumour* OR low-grade tumour* OR low grade tumour* OR brain cancer* OR brain tumour* OR brain tumour* OR brain metastasis OR brain metastases OR cerebral metastasis OR cerebral metastases OR intra-axial brain tumour* OR intra-axial brain tumour* OR intra axial brain tumour* OR intra axial brain tumour* OR intra-axial tumour* OR intra-axial tumour* OR intra axial tumour* OR intra axial tumour* OR intra-axial metastasis OR intra-axial metastases OR intra axial metastasis OR intra axial metastases OR intra-axial brain metastasis OR intra-axial brain metastases OR intra axial brain metastasis OR intra axial brain metastases OR diffuse glioma* OR diffuse astrocytoma* OR oligoastrocytoma* OR oligodendroglioma* OR cerebral tumour* OR cerebral tumour* OR metastasis OR metastases OR CNS neoplasm* OR neoplasm* OR brain

neoplasm* OR cerebral neoplasm* OR cerebral malignancy OR cerebral malignancies OR brain malignancy OR brain malignancies OR HGG OR LGG):ti,ab,kw

Modified QUADAS-2

**QUADAS-2 Risk of bias assessment**	
**Domain 1: Patient selection**	
**Signalling questions**	**Scoring with descriptions**
Are the study design and patient selection clearly described?	**Low risk:** Prospective/ retrospective. Patient selection and study design are clearly described, and no details are missing.<br>**Medium risk:** Details are derivable from context, but the description is unclear.<br>**High risk:** Case-control or other study design. Important details to reproduce the study are clearly missing.
Did the study avoid inappropriate exclusions?	**Low risk:** The study did not include inappropriate exclusion. Exclusions were clearly explained.<br>**High risk:** The study included inappropriate exclusion and/or exclusions that were not clearly explained.
Was a consecutive or random sample of patients enrolled?	**Low risk:** Consecutive/random sampling or can be derived from time range.<br>**High risk:** Other or not mentioned.
Was pathological confirmation of primary disease (and grade/mutation status) reliable and appropriate?	**Low risk:** Histopathological confirmation of disease and glioma grade/mutation status based on 2021 WHO CNS classification.<br>**Medium risk:** Radiological/clinical validation of disease and glioma grade/mutation status or histopathological confirmation based on previous WHO (or not specified) CNS classifications.<br>**High risk:** Not clearly described / not performed/ in case of radiological validation without clinical validation: insufficient or no follow-up imaging.
Was the first-line therapy clearly described and comparable to the standard?	**Low risk:** First-line therapy was clearly described. Dosages and administration schemes are described. First-line therapy choice, dosage and timing are comparable to standard and were administered as such.<br>**High risk:** Important details are missing, such as dosages or an administration scheme that is not described. First-line therapy choice, dosage, or timing differ from standard, e.g., standard therapy has not been completed.
**Domain 2: Index test**	
**Signalling questions**	**Scoring with descriptions**
Were the index test results interpreted without knowledge of the results of the reference standard?	**Low risk:** Index test results were interpreted without knowledge of the results of the reference standard (blinded).<br>**Medium risk:** This is not explicitly described, but based on the timing between the index tests and reference standard, a blind approach is highly probable.<br>**High risk:** Not described. Based on the timing between the index tests and reference standard, a blind approach is unlikely.

If a threshold was used, was it pre-specified?	**Low risk:** Threshold was specified a priori.<br>**High risk:** Threshold was not specified a priori.<br>**NA:** A threshold was not used.
**Domain 3: Reference standard**	
**Signalling questions**	**Scoring with descriptions**
Is the reference standard likely to correctly classify the target condition?	**Low risk:** Histopathological validation.<br>**Medium risk of bias:** Radiological validation according to RANO 2.0 criteria plus perfusion-weighted imaging or positron emission tomography *and* specialist clinical evaluation or radiological follow-up after three months or more.<br>**High risk:** Clinical validation only, or radiological validation with standard using insufficiently long follow-up, or reference standard is not described or other reference standard.
If the target condition was confirmed radiologically or clinically, was this confirmed by more than one observer? Is the assessment reliable and/or done according to guidelines?	**Low risk:** The target condition was confirmed by >1 observer with a board certificate in Neuroradiology. Assessment is done by multidisciplinary consultation.<br>**High risk:** The target condition was not confirmed by >1 observer with sufficient experience.
**Domain 4: Flow, timing, and analysis**	
**Signalling questions**	**Scoring with descriptions**
Was there an appropriate interval between therapy, index test(s), and reference standard? Was the interval between therapy, index test(s) and reference standard clearly described?	**Low risk:** There was an appropriate timing interval between therapy, index test(s) and reference standard(s). The interval was clearly described.<br>**Medium risk:** The interval can only implicitly be derived from the context.<br>**High risk:** There was an inappropriate interval between therapy, index test(s), and reference standard(s). The description is missing important details.
If quantitative analyses were performed, were the tests and results sufficient, fitting and clearly described?	**Low risk:** Performed tests were sufficient, fit the aim of the study and were clearly described. Reported results fit the aim of the study and were clearly described. All results were reported.<br>**Medium risk:** Minor information gaps regarding performed tests and results.<br>**High risk:** Major information gaps with performed tests and results. Tests and results did not fit the aim of the study or were not clearly described. Important results were omitted.<br>**NA:** Quantitative analysis was not performed.
Did all patients receive a reference standard, or, if applicable, were patients who did not receive a reference standard excluded from the analysis?	**Low risk:** All analysed patients received a reference standard. Patients who did not receive a reference standard were excluded from the analysis.<br>**High risk:** Not all patients received a reference standard, and those who did not were included in the analysis.
Did all patients receive the same reference standard? Were patients who received a different reference standard clearly described and explained?	**Low risk:** All patients received identical validation.<br>**Medium risk:** All patients received some adequate but not identical validation. The study clearly explains why one was opted for over the other.<br>**High risk:** Not all patients received histopathological or clinical validation, or it is unclear how comparable validations are.

Were all fitting patients included in the analysis or were withdrawals and exclusions explained?	**Low risk:** The analysis included all patients who fit the research aim. Withdrawals and exclusions were clearly explained.<br>**High risk:** The analysis did not include all patients who fit the research aim, and withdrawals and exclusions were not clearly explained.
If the reference standard is based on imaging, Are index tests and the reference standard performed in a way that allows comparison?	**Low risk:** Methodologies likely allow a comparison.<br>**High risk:** Methodologies likely do not allow a comparison

Calculation of risk of bias score per domain

Equation for score per domain: (total risk points domain / (N – N_{NA})) = R

Risk points: NA = 0, low risk = 0, medium risk = 1, high risk = 2

Score assessment: Low risk: $R < 0.5$, medium risk: $0.5 \leq R < 1.5$, high risk: $R \geq 1.5$

N = number of signalling questions, N_{NA} = number of signalling questions that are not applicable, R = risk of bias score

Applicability concerns assessment

Domains	Signalling questions	Scoring
**Patient selection**	Are there concerns that the included patients do not match the review question?	Yes/no
**Index test**	Are there concerns that the index test, its conduct, or its interpretation differ from the review question?	Yes/no
**Reference standard**	Are there concerns that the target condition, defined by the reference standard, does not match the review question?	Yes/no

Abbreviations: NA = not applicable, RANO = Response Assessment in Neuro-Oncology

*Notes (modifications):

- Signalling questions have been modified to better represent a real world application for APT-CEST in the clinical assessment of treatment response assessment in intra-axial brain tumours.
- To assess for the methodological reliability of the studies and the results, an ‘Analysis’ section was added to the ‘Flow and Timing’ domain to assess for possible heterogeneities.

Supplementary Table S1. Imaging parameters for APT-weighted image as MTRasym(3.5ppm)

Study	Hardware	Saturation approach	Saturation parameters	Readout	Acquisition and offset	B_0 correction and post-processing
Guo et al. 2022	Philips 3T	Pulse train	T_{sat}=830ms, t_p=200, t_d=10ms, B_1=2μT, n=4, DC_{sat}=95%	3D GRASE	6-offset protocol (S0, ±3, ±3.5, ±4 ppm)	WASSR B_0 map for B_0 correction
Hou et al. 2023	Philips 3T	MultiTransmit 4D	T_{sat}=2s, B_1=2μT	3D FSE	6-offset protocol (±3.1, ±3.5, ±3.9, and −1560 ppm)	3 acquisitions at ±3.5ppm to make map for B_0 correction
Huang et al. 2023	Philips 3T	Continuous wave	T_{sat}=2s, B_1=2μT	Unknown	Unknown	Intrinsic (CEST-Dixon) B_0 correction
Jiang et al. 2019	Philips 3T	Pulse train	T_{sat}=830ms, t_p=200ms, t_d=10ms, B_1=2μT, n=4, DC_{sat}=95%	3D GRASE	6-offset protocol (S0, ±3, ±3.5, ±4 ppm)	WASSR B_0 map for B_0 correction
Jiang et al. 2023	Philips 3T	Pulse train	T_{sat}=830ms, t_p=200, t_d=10ms, B_1=2μT, n=4, DC_{sat}=95%	3D GRASE	6-offset protocol (S0, ±3, ±3.5, ±4 ppm)	WASSR B_0 map for B_0 correction
Kroh et al. 2023	Siemens 3T	Pulse train	T_{sat}=830ms, t_p=200ms, t_d=10ms, B_1=2μT, n=4, DC_{sat}=95%	3D snapshot GRE	16 offsets (±4 (1), ±3.75 (2), ±3.5 (2), ±3.25 (2), and ±3 (1) ppm)	WASABI B_0 map for B_0 correction
Liu et al. 2020	General Electric 3T	Pulse train	T_{sat}=1.2s, t_p=400, t_d=0, B_1=1.5μT, n=3, DC_{sat}=100%	2D SE-EPI	Z-spectrum (M0, -1 to 1, ± 3 to ±4 with intervals of 0.25, ±1.5 to ±2.5 with intervals of 0.5, ±4.5, ±5, and ± 6)	Shifting minimum of Z-spectra to water resonance for B_0 correction
Ma et al. 2016	Philips 3T	Pulse train	T_{sat}=830ms, t_p=200ms, t_d=10ms, B_1=2μT, n=4, DC_{sat}=95%	3D GRASE	6-offset (S0, ± 3, ± 3.5, ± 4 ppm)	WASSR B_0 map for B_0 correction
Mehrabian et al. 2023	Philips 3T	Pulse train	T_{sat}=977.5ms, t_p=242.5ms, t_d=2.5ms, B_1=0.52μT / 2μT, n=4, DC_{sat}=95%	TFE	Z-spectrum (−5.9 to 5.9 ppm in 0.2 ppm increments, -780 ppm (3) and 2 after)	WASSR B_0 map for B_0 correction,<br>WASABI B_1 map for B_1 correction
Mehrabian et al. 2017	Philips 3T	Pulse train	T_{sat}=977.5ms, t_p=242.5ms, t_d=2.5ms, B_1=0.52μT / 2μT, n=4, DC_{sat}=95%	Single shot EPI	Z-spectrum (Z spectrum (−5.9 to 5.9 at 25 Hz increments))	Lorentzian line shape fitting for B0 correction
Paprottka et al. 2021	Philips 3T	Time-interleaved pTX	T_{sat}=2s, t_p=50ms, t_d=0ms, B_1=2μT, n=40, DC_{sat}=100%	3D FSE	6-offset protocol (± 3.5 ± 0.8, -1560ppm)	Intrinsic (CEST-Dixon) B_0 correction

Park et al. 2016_1	Unknown	Time-interleaved pTX	Unknown	3D GRE multishot EPI	Unknown	Unknown
Park et al. 2018	Philips 3T	Time-interleaved pTX	T_{sat}=2s, t_p=50, t_d=0ms, B_1=2μT, n=40, DC_{sat}=100%	3D TSE	6-offset protocol (± 3.5 ± 0.8, -1560ppm)	3 different echo times at +3.5ppm for B_0 correction
Park et al. 2016_2	Philips 3T	Time-interleaved pTX	T_{sat}=4.2s, t_p=70ms, t_d=70ms, B_1=1μT, n=30, DC_{sat}=50%	3D GRE multishot EPI	Z-spectrum (−5.0 to +5.0 at a step-size of 0.37)	Shifting minimum of Z-spectra to water resonance and 3 pool fitting for B_0 correction
Park et al. 2021	Philips 3T	Pulse train	T_{sat}=800ms, t_p=200ms, t_d=0ms, B_1=2μT, n=4, DC_{sat}=100%	3D GRASE	6-offset protocol (± 3.0, ± 3.5 (4x), and ± 4.0 ppm)	WASSR B_0 map for B_0 correction

Abbreviations: Acquisition and offset, presented as acquisition protocol (offsets (number of acquisitions at offset)); B1, radiofrequency pulse saturation amplitude; DCsat, saturation duty cycle (tp / (tp + td); FSE, fast spin echo; GRASE, gradient spin echo; GRE, gradient echo; n, number of pulse-element delay repetitions; ppm, parts per million; pTX, parallel transmit; SE-EPI, spin-echo echo planar imaging; T, Tesla; td, inter-pulse delay; TFE, turbo field echo; tp, pulse duration; Tsat, radiofrequency pulse saturation time; WASABI, simultaneous mapping of the water shift and B1; WASSR, water saturation shift referencing.

**Supplementary Table S2.** Characteristics of all included studies									
**Study and Design**	**Imaging method**	**tumour type and grade**	**N, sex (% female), age**	**Reference standard and TP/TR (number)**	**Intervention**	**Time between therapy and APT**	**ROI selection**	**APT signal TP/TR vs TRC and threshold/cutoff (c)**	**Diagnostic performance parameters (added) value of APT**
Guo et al. 2022; simulated prospective with data split	Multiparametric MRI (APT + T1 + T2 + FLAIR + GdT1)	High-grade glioma (WHO 3-4)	145; f (33.8%)<br><br>Age: 51.6 (mean)	HP and longitudinal clinico-radiological follow-up; TP (86)	RT/CRT following surgery	Median time: 257 (95-448) days (i), 183 (89–375) days (ii), 287 (190–542) days (iii)	Abnormalities on FLAIR co-registration	NA	Multi-MRI slice-level: Se (0.92); Sp (0.63); AUC (0.85)<br><br>Multi-MRI scan-level: Se (0.81); Sp (0.85); AUC (0.90)
Hou et al. 2023; prospective	APT and multi-parametric MRI (APT + 3D PCASL), ADC, 3D PCASL	High-grade glioma (WHO 3-4)	48; f (41.7%)<br><br>Age: 48.06 ± 11.73 (TP) and 50.29 ± 11.58 (TRC)	HP or clinico-radiological follow-up of >6 months; TR (31)	RT/CRT following surgery	Time: 326.18 ± 185.22 days (TRC); 263.20 ± 133.10 days (TP)	3 to 5 ROIs based on Gd-enhanced co-registered image	rAPTmax: c (2.25%)	APT:<br>Se (0.903); Sp (0.824); AUC (0.911)<br><br>PCASL + APT:<br>Se (0.935); Sp (0.941); AUC (0.951)
Huang et al. 2023; prospective	APT- and multi-parametric MRI (APT+QSM+PCASL), QSM, PCASL	High-grade glioma (WHO 3-4)	39; f (30.8%)<br><br>Age: 54.9 ± 16.2 (TR) and 57.4 ± 10.1 (TRC)	HP or integrated clinical pathologic results; TR (22)	CRT following surgery	Median time: 286 days (156–468)	ROIs based on Gd-enhanced tumour contour	APTmean (p=0.023): 2.24 ± 0.71 vs 1.66 ± 0.82<br><br>APTmax (p=0.006): 2.18 ± 0.94 vs 1.37 ± 1.04<br><br>APT90* (p=0.002): 3.5 ± 0.67 vs 2.83 ± 0.69	APT:<br>AUC (0.74)<br><br>APT + QSM + PCASL:<br>AUC (0.89)

Jiang et al. 2019; retrospective	APT	High-grade glioma (WHO 3-4)	21; f (23.8%)<br><br>Age 54.6 ± 17	HP; TR (18)	CRT following surgery	Median time: 353 days (range 43 – 1311)	2 to 5 ROIs based on APTw and other MRI acquisitions (Gd enhanced)	APTmean (p<0.05): 2.71 ± 0.91% vs 1.24 ± 0.29%; c (1.79%)	APT:<br>Se (0.944); Sp (1.0)
Jiang et al. 2023; retrospective	APT and multi-parametric MRI (APT + T1 + T2 + FLAIR, GdT1)	High-grade glioma (WHO 3-4)	86; f (38.4%)<br><br>Age: 50.4 ± 12.4 (TP) and 55 ± 13.1 (TRC)	HP or clinico-radiological diagnosis using to RANO; TP (60)	RT/CRT ± surgery	Median time: 185 days (range 18–3655)	FLAIR abnormalities on co-registered image	NA	APT:<br>Se (0.706); Sp (0.962); AUC (0.878)<br><br>Multi-parametric MRI:<br>Se (0.85); Sp (1); AUC (0.925)
Kroh et al. 2023; prospective	APT and MT (CEST)	Diffuse glioma (WHO 2-4)	27; f (unknown)<br><br>Age: unknown	Clinico-radiological follow-up for median 9.2 months using RANO; TP (19)	RT/CRT ± surgery	Time: 4-6 weeks following RT completion	ROI based on T1 and FLAIR	APT_{asym} signal could not distinguish PsP from TP	Unknown
Liu et al. 2020; Prospective	APT and multi-parametric MRI (APT + ASL), ASL	High-grade glioma (WHO 3-4)	30; f (53.3%)<br><br>Age: 44.3 ± 13.5	HP or clinico-radiological follow up > 6 months using RANO; TR (16)	RT/CRT following surgery	0.9 ± 17.8 days (TP) and 27.4 ± 31.9 days (TRC)	2 to 5 ROIs based on Gd enhancing area	APTmean (p<0.001): 1.56 ± 1.14% vs -0.44 ± 1.34%	APT:<br>Se (0.875); Sp (0.786); AUC (0.87)<br><br>APT + ASL:<br>Se (0.938); Sp (0.929); AUC (0.93)
Ma et al. 2016; Unknown	APT	Low- and high-grade gliomas (WHO 1-4)	32; f (34.4%)<br><br>Age: 56.5 [22-78]	Clinico-radiological follow up > 6 months using RANO; TR (20)	CRT ± prior surgery	3 months (range 1-12)	3 to 5 ROIs based on Gd enhancing area	APTmean* (p<0.001): 2.75 ± 0.42% vs 1.56 ± 0.42%; c (2.42%)<br><br>APTmax (p<0.001): 3.29 ± 0.61% vs 1.95 ± 0.44%; c (2.54%)	APT:<br>Se (0.85); Sp (1.0); AUC (0.98)

Mehrabian et al. 2017; prospective	APT, MTR_{NOE}, MT	Cerebral metastases	16; f (62.5%)<br><br>Age: 63.5 [39-73]	HP or clinico-radiological follow-up; TP (5)	SRS	12.6 months	ROI based on Gd-enhanced 3D axial-weighted imaging	<u>APTmean</u> (p=0.89): −0.6 ± 1.0 vs −0.7 ± 1.0	<u>APT</u>:<br>Se (0.8); Sp (0.36); AUC (0.436)
Mehrabian et al. 2023; prospective	APT, MTR_{rNOE}, MTR_{Amide}, $AREX_{Amide}$, $AREX_{rNOE}$	Cerebral metastases	75; f (68%)<br><br>Age: 60 [55-65]	HP and clinico-radiological follow up > 6 months; TR (30)	SRS	>3 months (516 ± 442 days)	ROI based on whole contrast-enhancing lesion on T1Gd	<u>APTmean</u> (p=0.23): 1.7 ± 1.5 vs 1.2 ± 2.0; c (1.4)	<u>APT</u>:<br>Se (0.58); Sp (0.67); AUC (0.58)
Paprottka t al. 2021; retrospective	Multi-parametric MRI (APT + T1 + FLAIR + T2 + DSC ± FET-PET)	Low- and high-gade gliomas (WHO 1-4)	74; f (44.6%)<br><br>Age: 54.91 ± 12.2	HP or long term clinico-radiological follow-up after 3 months using RANO; TR (57)	RT following surgery	102 days	ROI based on hot spot delineation on co-registered images	NA; c (1.79%)	<u>Multi-parametric MRI</u>:<br>Se (0.95); Sp (0.45)<br><br><u>Multi-parametric MRI + FET-PET</u>:<br>Se (0.91); Sp (0.701); AUC (0.85)
Park et al. 2016_1; retrospective	APT	High-grade glioma (WHO 3-4)	21; f (42.9%)<br><br>Age 54 ± 12.22 (TP) and 50.33 ± 14.6 (TRC)	HP or clinico-radiological follow-up; TP (13)	RT/CRT following surgery	10-45 days	ROIs encompassing entire contrast enhancing solid tumour on co-registered T1-weighted image.	<u>APT90</u> reader 1: 2.7% ± 0.8 vs 0.9% ± 0.8; c (1.90%)<br><br><u>APT90</u> reader 2: 2.8% ± 1.4 vs 0.8% ± 0.9; c (1.98%)	<u>APT</u>:<br>AUC (0.90)
Park et al. 2016_2; retrospective	APT and multi-parametric MRI (APT + DSC + CE-T1WI), DSC, CE-T1WI	GBM (WHO 4)	65; f (53.8%)<br><br>Age: 54.3 (24-77)	HP or clinico-radiological follow-up >6 months; TR (37)	CRT following surgery	47.5 weeks	ROI based on contrast-enhancing lesion on co-registered image	<u>APT90</u>: 3.87 ± 1.72 vs 1.38 ± 1.14; c (2.88%)	<u>APT</u>:<br>AUC (0.89)<br><br><u>Multi-parametric MRI</u>:<br>AUC (0.97)

Park et al. 2018; retrospective	APT and MET-PET	Diffuse glioma (WHO 2-4)	43; f (45.8%)<br><br>Age: 52.1 (32-73)	HP or clinico-radiological follow-up from 20 to 49 months using RANO; TR (38)	RT/CRT	150 days (HGG) and 608 days (LGG)	ROIs based on contrast-enhanced T1, hyperintense lesions on T2, and hyperintense APTw and TNR signal.	APT90 reader 1: c (1.79%)<br><br>APT90 reader 2: c (1.96%)<br><br>APTmax*: c (2.03%)	APT:<br>Se (0.862); Sp (0.857); AUC (0.88)
Park et al. 2021; retrospective	APT and multi-parametric MRI (APT + DWI + DSC + DTI), DWI, DTI, DSC	Diffuse glioma (WHO 2-4)	36; f (50%)<br><br>Age: 52.3 ± 13.7 (TR); 57.1 ± 17.8 (TRC)	HP and clinico-radiological follow-up at 2-3 month intervals; TR (25)	RT/CRT following surgery	77.7 ± 141.3 (TR) and 31.7 ± 30.1 (TRC)	ROI based on APT image and exclusion of necrotic and cystic lesions by T2 and post-contrast T1	APTmean (p=0.009): 3.18% vs 1.77%; c (2.11%)	APT:<br>Se (0.84); Sp (0.727); AUC (0.87)<br><br>Multi-parametric MRI:<br>Se (0.92); Sp (0.909); AUC (0.92)

Abbreviations: i, training dataset; ii, validation dataset; iii, testing dataset; age, age presented as either mean ± range, mean (lowest – highest) or median [1st – 3rd quartile]; APT, amide proton transfer defined as signal at magnetisation transfer ratio asymmetry analysis at 3.5 parts per million; APT90, 90% histogram value APT; APTmax, max histogram value of APT; APTmean, mean histogram value of APT; AREXAmide, apparent exchange-dependent relaxation of amide; AREXrNOE, apparent exchange-dependent relaxation of relayed nuclear Overhauser effect; AUC, area under the curve; c, cutoff value where signal > c is TR and signal < c is TRC; CEST, chemical exchange saturation transfer; CE-T1WI, contrast-enhanced T1-weighted image; clinico-radiological follow-up, thorough clinical and radiological follow-up; CRT, chemoradiotherapy; DSC, dynamic susceptibility contrast; DTI, diffusion tensor imaging; DWI, diffusion-weighted imaging; ; f, percentage of female patients; FET-PET, fluor-ethyl-tyrosine positron emission tomography; FLAIR, fluid attenuated inverse recovery MRI; GBM, glioblastoma; GdT1, Gadolinium-enhanced T1-weighted image; HGG, high-grade glioma; HP, histopathological assessment through biopsy/resection; LGG, low-grade glioma; MET-PET, methionine positron emission tomography; MT, magnetisation transfer; MTRAmide, magnetisation transfer ratio of amide (+3.5ppm); MTRNOE, magnetisation transfer ratio of nuclear Overhauser effect (-3.5ppm); MTRrNOE, magnetisation transfer ratio of relayed nuclear Overhauser effect; Multi-(parametric) MRI, APT imaging in combination with other conventional or advanced MRI imaging; N, number of scans analyzed; NA, not applicable; (PC)ASL, (pseudocontinuous) arterial spin labelling; RANO, response assessment in neuro-oncology criteria; rAPTmax, relative APT-weighted max signal (APTtumour – APTnawm); ROI, region of interest; RT, radiotherapy; Se, sensitivity; Sp, specificity; SRS, stereotactic radiosurgery; T1, T1-weighted MRI; T2, T2-weighted MRI; TP, true progression; TR, true recurrence; WHO, World Health Organization glioma classification.

Footnotes:

- If more than one histogram parameter was assessed, the best diagnostic performance was reported for APT-CEST imaging.
- If 2 or more readers assessed the APT-CEST image, the ROI that gave the best diagnostic performance was selected.
- For Mehrabian 2023, the results of B1 (amplitude) of 2µT (micro-Tesla) were used.

* best performing parameter
